# Changes in Cerebral Blood Flow after Erenumab Treatment in Good and Non-Responders—A Pilot Study of Migraine Patients

**DOI:** 10.3390/jcm10112523

**Published:** 2021-06-07

**Authors:** Magdalena Nowaczewska, Marcin Straburzyński, Grzegorz Meder, Wojciech Kaźmierczak

**Affiliations:** 1 Department of Otolaryngology, Head and Neck Surgery, and Laryngological Oncology, Ludwik, Rydygier Collegium Medicum in Bydgoszcz, Nicolaus Copernicus University, M. Skłodowskiej-Curie 9, 85-090 Bydgoszcz, Poland; 2 Athleticomed—Pain & Sport Injury Centre with Headache & Migraine Treatment Divsion, Fordonska 144, 85-357 Bydgoszcz, Poland; 3 Headache Clinic—Terapia Neurologiczna ‘Samodzielni’, Mochnackiego 10, 02-042 Warsaw, Poland; marcinstraburzynski@gmail.com; 4 Department of Interventional Radiology, Jan Biziel University Hospital No. 2, Ujejskiego 75 Street, 85-168 Bydgoszcz, Poland; grzegorz.meder@gmail.com; 5 Department of Sensory Organs Examination, Faculty of Health Sciences, Collegium Medicum in Bydgoszcz, Nicolaus Copernicus University, M. Skłodowskiej-Curie 9, 85-090 Bydgoszcz, Poland; wojciech.kazmierczak@umk.pl

**Keywords:** CGRP antibody, erenumab, cerebral blood flow, transcranial Doppler, migraine, headache, medication overuse, MOH, efficacy, outcome

## Abstract

Erenumab showed efficacy in migraine prevention, however we cannot identify which patients to treat by predicting efficacy response. The aim of this study was to compare changes in cerebral blood flow (CBF) reflected by transcranial Doppler (TCD) in erenumab good responders (GR) and non-responders, in order to identify a parameter that could predict the treatment response. In this study, migraineurs treated with erenumab underwent clinical and TCD evaluations before and 6 weeks after the treatment, including data on migraine type, monthly migraine days (MMD), medication overuse headache (MOH) presence, mean blood flow velocity (Vm) and pulsatility index (PI) in cerebral arteries (CA). GR were defined as reporting ≥50% reduction in MMD. Thirty women were enrolled, of mean age 40.53 years, 20 with chronic migraine, 14 with MOH, and 19 were GR. Baseline Vm values in right CA and basilar artery (BA) were significantly lower in GR as compared with non-responders. Vm values in all arteries significantly increased after the treatment as compared with corresponding baseline values, but only in GR. A significant negative correlation was observed between baseline Vm in right CA and treatment effectiveness. Baseline Vm in right CA and basilar artery is reduced in erenumab GR as compared with non-responders. This asymmetry normalizes after the treatment with significant Vm increase in CA which may reflect CBF increase in GR only. Lower baseline Vm in right CA may predict erenumab efficacy; however, these results should be replicated in a larger cohort.

## 1. Introduction

Calcitonin gene-related peptide (CGRP) plays an important role in the pathophysiology of migraine and anti-CGRP monoclonal antibodies (mAbs) have been developed and showed efficacy in the prevention of migraine attacks [1]. Among four already available mAbs Erenumab (Novartis/Amgen) was the first in class approved by drug agencies and the only one blocking the CGRP receptors. Erenumab is a fully human monoclonal antibody, administered subcutaneously once monthly and has been shown to be effective for migraine prevention [2,3]. Nevertheless, together with excitement for the clinical trials success, some concerns have been raised for the potential harmful effect of blocking CGRP-mediated vasodilation [4,5]. Up to now, there has been a lack of studies regarding the influence of anti-CGRP mAbs on cerebral blood flow (CBF) while those already published remain conflicting and inconclusive [5,6,7,8,9,10]. Moreover, we are not able to select which patient is a good candidate to treat by predicting efficacy response. Transcranial Doppler ultrasonography (TCD) makes it possible to assess blood flow in the intracranial arteries non-invasively. TCD parameters are influenced both by changes in cerebral vessels diameters and CBF, moreover CBF correlates with the flow velocity in these vessels [11]. Therefore, the question arises as to whether erenumab induce cerebral hemodynamic changes which could be detected by TCD. The aim of this pilot study was to compare changes in CBF reflected by TCD in erenumab good responders and non-responders, in order to identify the parameters that could predict the response to the treatment.

## 2. Materials and Methods

This observational, retrospective case-control study involved migraineurs treated at our Headache Centre and receiving the clinical indication for migraine prevention with erenumab (Aimovig, Novartis Europharm Limited) according to the available guidelines and who agreed to participate in this study. All patients received the same dose of erenumab (70 mg per month).

The inclusion criteria included migraine with or without aura according to the International Classification of Headache Disorders (ICHD-3), age 18 to 65 years, at least 4 migraine days for a month [12].

The exclusion criteria included inadequate temporal windows, stenosis of the intracranial arteries, hemodynamically significant stenosis of the internal carotid arteries, atrial fibrillation, cardiovascular disease and other serious somatic or psychiatric disorders, treatment with migraine prophylactic drugs when on erebumab treatment and on the day of first TCD assessment. Patients with a headache on the day of examination or patients using painkillers or triptans on the day of examination were asked to reschedule the visit. Coffee, tea, cocoa, cola, energetic drinks and alcohol were not allowed for 6 h before scheduled examination. Patients were taught erenumab self-administration and received erenumab every 4 weeks by subcutaneous injection at the dose of 70 mg. All patients received two erenumab injection during the study period.

All patients underwent the clinical and TCD evaluations at baseline before erenumab injection and between 6 and 7 weeks of treatment (6–7 weeks after the first erenumab injection, 2–3 weeks after second erenumab injection). Data on migraine onset age, migraine type, monthly migraine days (MMD), monthly headache days (MHD), acute medication days (AMD), type of acute medication used, headache intensity using a numerical scale (numeric rating scale, NRS), headache burden using the Headache Impact Scale (HIT-6) were collected before and after the treatment. Early treatment efficacy was assessed based on the patients’ last month of receiving mAbs, between 6 and 7 weeks of treatment. Based on treatment efficacy we divided patients into two groups: good responders and non-responders. Patients, who reported a good effect of treatment (≥50% reduction in MMD), were defined as good responders. The remaining patients were defined as non- responders. Then we compared TCD parameters and clinical data between those two groups.

Patients were also classified as suffering from chronic (CM) or episodic (EM) migraine according to the ICHD-3 [12]. Patients with all degrees of medication overuse headache (MOH) were included. MOH was defined according to the ICDH-3 [12].

All the procedures were approved by the Local Ethics Committee of the Ludwik Rydygier Collegium Medicum in Bydgoszcz. The subjects gave their informed consent before the start of any procedure.

TCD examinations were performed with a commercially available TCD apparatus: Nicolet Sonara transcranial Doppler system (producer Viasys Healthcare) and a 2 MHz probe. The study was performed in a quiet room with the subjects lying in a comfortable supine position, after 10 min rest. Intracranial arteries were insonated through the temporal and transforaminal windows using standardized protocol, by the technique described by Aaslid [13]. After finding the temporal window, the middle cerebral artery (MCA) was identified, then posterior cerebral artery (PCA). Vertebral arteries (VA) and basilar artery (BA) were assessed using the suboccipital window. Because of the usual anatomic course of anterior cerebral artery, the TCD assessment of this vessel is difficult and accuracy of mean velocity (Vm) measurement is small so we excluded ACA flow parameters from this study [13,14]. The probe was positioned in such a way as to obtain the highest possible velocity of the tested vessel. Vm and Gosling’s pulsatility index (PI) were measured and recorded at a 54–56 mm depth in both MCAs, at 55–65 mm in both PCAs, at 50–70 mm in both VAs, and at 80–100 mm at BA. Gosling’s PI was calculated as the difference between Vmax and Vmin, divided by the mean velocity. Only measurements with the best signal-to-noise ratio were used, and the highest values for CBF velocities were selected for analysis. TCD tests were always performed by the same physician experienced in the field of neurosonology (MN). Before every TCD examination blood pressure and heart rate were measured.

The results are presented as arithmetic means and their standard deviations (SD). To compare TCD parameters between right and left hemisphere arteries, according to variable distribution, either *t*-test or non-parametric Mann–Whitney U tests were adopted. A paired *t*-test or Wilcoxon test were used to test the significance of differences between two measurement points in the same group and analysis of variance (ANOVA) for responses between the groups. Correlation between TCD parameters and physiologic variables affecting flow velocities, such as age, duration of migraine, MMD or AMD were tested using Pearson correlation test, while for testing the correlations between TCD parameters and HIT/NRS scores Spearman correlation was used. The Shapiro–Wilk test was used to check for normality. Calculations were performed using Statistica 7 (StatSoft, Kraków, Poland) software. Statistical significance was set at *p* ≤ 0.05, two-tailed.

## 3. Results

Thirty three patients were enrolled for this study, all were women. Among them, thirty were finally analyzed after excluding three patients: two with inaccurate temporal bone window, one with MCA stenosis. The mean age was 40.53 ± 11.09 years (range: 20 to 65 years). Ten patients were diagnosed with episodic migraine, while 20 patients with chronic migraine. Five patients were diagnosed with migraine with aura. Fourteen patients were additionally diagnosed with MOH. The duration of migraine was 20.9 years (range 5 and 40 years). 19 patients had >50% responder rate after erenumab treatment. There was a statistically significant decrease in MMDs, MHDs, AMDs, NRS and HIT score after the treatment compared with baseline in both groups. Patients were overusing mostly triptans, combination codeine medicines, paracetamol or ibuprofen. The characteristics of the patients depending on treatment efficiency are presented in Table 1.

**Figure 1:**
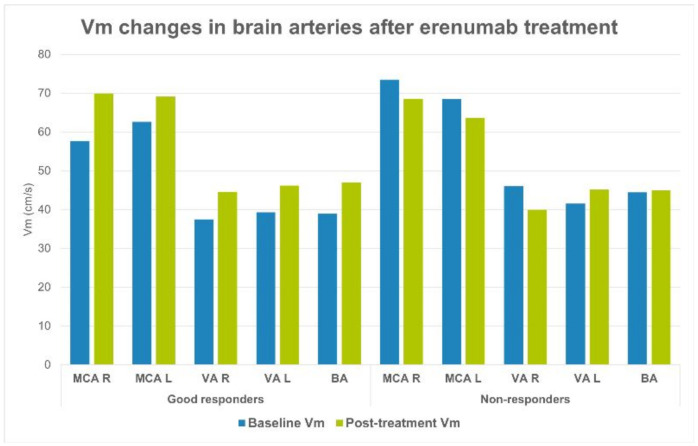
Vm changes after erenumab treatment in brain arteries. Abbreviations: Vm—mean flow velocity, MCA—middle cerebral artery, ACA—anterior cerebral artery, BA—basilar artery, R—right, L—left.

There were no significant differences regarding Vm and PI between right and left hemisphere in all examined arteries before the treatment and after the treatment (whole group, good responders and non-responders). Baseline Vm value in MCA R, VA R and BA were significantly lower in the good responders group compared with non-responders group. Vm values in both MCAs, VAs and in BA after the treatment significantly increased as compared with corresponding baseline values, but only in a good responders group. In non-responders, Vm decreased after the treatment, however, this difference was not statistically significant. The Vm in PCAs did not change during the erenumab treatment in both groups. There were no PI differences between groups before and after the treatment. In the non-responders group PI significantly increased in some arteries after the treatment, while there were no changes in good responders group regarding PI (Table 2 and Table 3, Figure 1). Blood pressure and heart rate values did not significantly differ between groups before and after the treatment.

There was a significant negative correlation between age, migraine duration and baseline Vm in both MCAs. Moreover, a correlation was observed between baseline Vm in right MCA and right VA and treatment effectiveness: with the Vm increase, the chance for >50% responder rate decreased (−0.47, *p* < 0.009; −0.37, *p* < 0.046 respectively). No correlation was found between NRS, HIT-6, MMD, MHD, AMD and Vm in any of examined arteries.

## 4. Discussion

The most important and interesting finding of this study is that erenumab good responders had significantly lower baseline Vm in right brain arteries and BA as compared to non-responders which correlated with treatment effectiveness. Moreover, Vm values significantly increased after the treatment in the good responders group only, remaining unchanged in non-responders. Why do good responders differ from non-responders regarding baseline CBF parameters and what can be responsible for increased flow velocity in brain arteries after erebumab treatment?

To further discuss this phenomenon it should be emphasized that cerebral arteries have their own muscle tone and respond with an active contraction to the increase in transmural pressure, thus differing from arteries of other localisation. It is known that cerebral arteries are in a state of partial contraction, which allows them to change their diameter to regulate CBF. Because blood flow is related to the diameter of the vessel, even a slight increase in the smooth muscle tone of the vessel will reduce its diameter and will lead to flow disturbances [15,16]. Assuming that the diameter of the vessel does not change, velocity correlates with CBF—therefore an increase in CBF increases blood flow velocity. Thus, in our study, an increase in velocity in good responders could be associated with an increase in CBF. However, another explanation could be that there was a change in the MCA, VA and BA diameters in response to erenumab. As CGRP has a direct vasodilatory effect, a part of erenumab’s action could be preventing vasodilation or theoretically even inducing vasoconstriction [17]. This mechanism may lead to arteries shrinkage, which induce an increase in the blood flow velocity in those vessels. Nevertheless, this theory is hard to prove as erenumab showed no direct contractile or relaxant effects in previous studies, so is probably not associated with vasoactive properties [8]. Moreover, even if CGRP dilates cerebral arteries, the effect is so small that it is unlikely to be the only mechanism of CGRP-induced migraine [18]. A number of studies confirmed that erenumab inhibits CGRP-induced vasodilatory responses in human arteries; however, it does not modify cerebral vasomotor reactivity and brachial flow-mediated dilation [6,9]. Assuming that erenumab does not change the cerebral arteries’ diameter, the only possible explanation of Vm increase in good responders group after erenumab treatment in our study could be a CBF increase. A small number of studies accesses CBF after erenumab treatment. Altamura et al. did not find a difference regarding Vm in MCA and PCA 2 weeks after erenumab injection. However, they did not provide information about medication overuse in examined patients [6]. Ziegler et al. in functional magnetic resonance imaging (fMRI) study detected no global alteration of CBF after erenumab treatment, but they observed a decrease in relative CBF in the left frontal white matter [10]. It is important to notice that patients with MOH were excluded from this study. Although little is known about CBF in migraine, there is evidence that CBF changes are associated with the different clinical courses of migraine, especially remission and progression groups [19].

In our study, together with Vm increase after erenumab treatment, we did not observe a change in PI. The value of PI, in some circumstances, illustrates the resistance of a vascular bed (supplied by a given artery) when examined by TCD. There is a linear relationship between changes in PI and intracranial pressure (ICP). A decrease in PI may result both from lowered ICP and from vascular dilatation. However, it must be noted that the usefulness of PI regarding CBF assessment is small as it may be influenced by many other factors [20,21,22]. As our good responders presented only with Vm increase and unchanged PI it may suggest that vessels diameter was also unchanged so the Vm increase reflects CBF increase. Although no PI changes were noted in good responders, we do observed an increase of PI in non-responders after erenumab treatment. As Vm was stable, a PI increase might be a result of increase resistance in microcirculation in this group.

There is another possible explanation for flow velocity increase after erenumab treatment. As this effect was observed in good responders only, it might be linked with reduced acute medication intake after erenumab treatment in this group. For example it is known that CBF decrease significantly after risatriptan administration due to vasoconstriction. However, this effect is short lasting and CBF recovers to baseline after 80 min [23]. Nevertheless, CBF in migraineurs overusing triptans was never studied. One cannot exclude the possibility that increased triptans intake may continuously reduce CBF by vasoconstriction and triptans cessation may inversely increase CBF. The blood flow velocity (BFV) increase was proven after ergotamine overuse—which is an anti-migraine drug similar to the triptans vasoconstricting effect. Savrun at al. measured CBF by means of TCD in patients suffering from probable MOH. They discovered that patients with probable ergotamine overuse had increased BFV in MCA and BA probably via vasoconstriction [24]. Contrary to these findings, patients with probable analgesic-overuse headache had lower mean BFV of all the vessels than control group but no statistical significance was found. They concluded that analgesic overuse results in a functional disorder of neuronal receptor and neurovascular reflexes and may cause a reduction of intracerebral vessel tone, leading to vasodilatation [24].

Another question to answer is why we observed baseline Vm asymmetry in right arteries between GR and NR as well as why Vm was associated with outcome only in the right cerebral arteries and BA? Is it only a matter of small study group size? Interestingly, in a functional imaging study, Ziegler et al. found that erenumab leads to a decrease in activation in the right thalamus, right middle temporal gyrus, right lingual gyrus and left operculum. However, when contrasting responders and non-responders they found a significant reduction of hypothalamic activation after erenumab injection in responders only suggesting that erenumab may have an additional central effect. They suggested that erenumab may cross the blood–brain barrier in very small, but effective concentrations in some patients with a direct hypothalamus activation [10]. A number of other authors reported asymmetry in CBF between hemispheres during migraine attack as well as in the interictal period [25,26,27,28,29]. However, it should be noted that studies assessing cerebral hemodynamics in migraine reported inconsistent findings. In a MRI study, Coppola at al. found a correlation between changes in the right thalamus and the days from the last attack, also higher thalamic fractional anisotropy values were noted during the interictal period and were normalized during an attack [30]. In a SPECT (Single Photon Emission Computer Tomography) study Mirza et al. revealed interhemispheric asymmetry of the brain during headache-free intervals in migraineurs suggesting an impaired regional cerebral vascular autoregulation [31]. Another study assessing CBF in migraine patients found that headache frequency was positively correlated to CBF in the right anterior inferior temporal gyrus [32]. Lee at al. found that side-to-side asymmetry of CBF velocity is not constant but a dynamic factor throughout the course of the migraine. They hypothesized that the side with higher CBF velocities may indicate regional neurovascular activation in migraineurs. Further investigation of the clinical significance of asymmetric CBF may be warranted [19]. In our study, good responders had significantly lower Vm in right arteries and BA before erenumab treatment as compared with non-responders group, which was reversed after erenumab treatment. This findings may suggest lower baseline CBF in good responders group. Additionally, we observed a negative correlation between baseline Vm in right MCA and right VA and treatment effectiveness: with the Vm increase, the chance for >50% responder rate decreased. It suggest that reduced Vm in right cerebral arteries before starting erenumab treatment might be a good parameter to predict the outcome, however this finding needs future studies with larger cohorts.

Surprisingly, we found no differences regarding CBF between migraineurs with or without MOH, as well as between episodic and chronic migraine before and after erenumab treatment. One of the possible explanation may be a fact that we included patients with high-frequency episodic migraine.

It is worth noting that contrary to Vm increase in good responders, a Vm decrease was observed in non-responders after the treatment as compared with baseline parameters. This Vm decrease, although not statistically significant, seems interesting and needs to be deepened in larger studies.

The main limitation of this study is the small group of patients. Another may be a too early time point for the efficacy evaluation, as some patients may have responded to the treatment later. Besides, it should be remembered that TCD measures only the flow velocity and not the absolute CBF value. The correlation between CBF and flow velocity is variable. Cerebral blood velocity is an adequate surrogate of absolute flow only if the insonated vessel maintains constant vessel diameter across time and experimental conditions. Blood flow velocity is further influenced by several factors, including arterial blood pressure, ICP, hematocrit, PaCO2 and the status of autoregulation, thus making a direct comparison of flow velocity and CBF difficult. Another limitation of any migraine study is that in high-frequency episodic or chronic migraine, even when the examinations are performed outside the headache phase, it cannot be excluded that patients are in the early prodromal or late postictal phases.

## 5. Conclusions

Baseline Vm in right MCA, VA and BA is reduced in erenumab good responders as compared with non-responders. This interhemispheric asymmetry normalizes after the treatment as a result of Vm increase in the good responders group only. Whether it is an effect of acute medication cessation or erenumab itself remains unknown. Lower baseline Vm in right cerebral arteries and BA may predict erenumab efficacy; however, due to small numbers of patients, these results need to be replicated in larger cohorts.

## Figures and Tables

**Table 1:** Clinical characteristics of migraine patients treated with erenumab depending on treatment efficacy.

Parameter	Good Responders ≥50% RR	Non-Responders <50% RR	*p*-Value
	*N* = 19	*N* = 11	
Age (mean ± SD)	42.05 ± 13.18	37.91 ± 5.70	0.24
BMI, kg/m^2^	23.6	22.5	0.314
Duration of migraine (years)(mean ± SD)	21.84 ± 10.51	19.27 ± 7.24	0.44
Type of migraine: Episodic Chronic	7 (36.84) 12 (63.16)	3 (27.27) 8 (72.73)	0.7
Migraine with aura, *n* (%)	3 (15.79)	2 (18.18)	1
MOH, *n* (%)	8 (42.11)	6 (54.55)	0.78
MMD-Baseline MMD-Post-treatment	11.26 ± 5.19 3.21 ± 2.86	11.91 ± 4.11 9.91 ± 5.05	0.56 0.001
*p*-value	0.0001	0.009	
MHD-Baseline MHD-Post-treatment	18.32 ± 7.93 5.74 ± 4.76	20.18 ± 7.24 15.73 ± 6.51	0.52 0.0004
*p*-value	0.00005	0.006	
AMD-Baseline AMD-Post-treatment	13.42 ± 6.27 3.68 ± 2.98	16.18 ± 7.48 10.18 ± 4.73	0.32 0.001
*p*-value	0.0001	0.02	
NRS-Baseline NRS-Post-treatment	8.32 ± 1.06 5.37 ± 2.93	8.55 ± 1.21 7.36 ± 1.12	0.61 0.02
*p*-value	0.0004	0.01	
HIT-6-Baseline HIT-6-Post-treatment	62.53 ± 5.31 46.63 ± 8.39	63.45 ± 5.01 59.00 ± 7.14	0.81 0.0009
*p*-value	0.0001	0.04	
Mood disorders, *n* (%)	5 (26.32)	5 (45.45)	0.43
Thyroid disease, *n* (%)	4 (21.05)	3 (27.27)	1
Oral contraceptives, *n* (%)	3 (15.79)	2 (18.18)	1
Acute medication used Triptans Codeine Ibuprofen or paracetamol	9 (70.9) 7 (36.8) 1 (5.3)	6 (54.5) 2 (18.18) 3 (27.3)	0.82 0.06 0.11

Abbreviations—SD = standard deviation, BMI = body mass index, NRS—numeric rate scale, HIT-6 = headache impact test-6, MOH—medication overuse headache, MMD—monthly migraine days, MHD—monthly headache days, AMD—monthly medication days.

**Table 2:** The mean velocity (Vm) values before and after erenumab treatment depending on treatment efficacy.

Parameters	Good Responders (≥50% RR)	Non-Responders (<50%RR)	*p*-Value
	*N* = 19	*N* = 11	
Baseline Vm, MCA R (cm/s)	57.8	73.45	0.008
Post-treatment Vm, MCA R (cm/s)	69.93	68.56	0.83
*p*-value	0.0003	0.18	
Baseline Vm, MCA L (cm/s)	662.6	68.5	0.17
Post-treatment Vm, MCA L (cm/s)	69.11	63.67	0.11
*p*-value	0.016	0.27	
Baseline Vm, VA R	37.5	46.05	0.02
Post-treatment Vm, VA R	44.61	39.94	0.17
*p*-value	0.02	0.07	
Baseline Vm, VA L	39.3	41.58	0.55
Post-treatment Vm, VA L	46.21	45.16	0.82
*p*-value	0.002	0.4	
Baseline Vm, BA	38.93	44.42	0.04
Post-treatment Vm, BA	47	44.95	0.3
*p*-value	0.0002	0.85	

Abbreviations: RR—responders rate, Vm—mean velocity, MCA—middle cerebral artery, VA—vertebral artery, BA—basilar artery, R—right, L—left.

**Table 3:** The PI values before and after erenumab treatment depending on treatment efficacy.

Parameters	Good Responders (≥50% RR)	Non-Responders (<50%RR)	*p*-Value
	*N* = 19	*N* = 11	
Baseline PI, MCA R	0.78	0.84	0.18
Post-treatment PI, MCA R	0.5	0.87	0.62
*p*-value	0.32	0.35	
Baseline PI, MCA L	0.78	0.78	0.48
Post-treatment PI, MCA L	0.8	0.87	0.17
*p*-value	0.58	0.04	
Baseline PI, VA R	0.81	0.76	0.31
Post-treatment PI, VA R	0.83	0.86	0.64
*p*-value	0.64	0.002	
Baseline PI, VA L	0.78	0.76	0.73
Post-treatment PI, VA L	0.78	0.82	0.46
*p*-value	0.81	0.23	
Baseline PI, BA	0.8	0.76	0.41
Post-treatment PI, BA	0.85	0.88	0.58
*p*-value	0.17	0.01	

Abbreviations: PI—Gosling’s pulsatility index, RR—responders rate, Vm—mean velocity, MCA—middle cerebral artery, VA—vertebral artery, BA—basilar artery, R—right, L—left.

## Data Availability

The data presented in this study are available on request from the corresponding author. The data are not publicly available due to privacy restrictions.
